# Cost-effectiveness of antibiotic prophylaxis in elective cesarean section

**DOI:** 10.1186/s12962-018-0168-x

**Published:** 2018-12-18

**Authors:** Markus H. Jansson, Yang Cao, Kerstin Nilsson, Per-Göran Larsson, Lars Hagberg

**Affiliations:** 1 Department of Obstetrics and Gynecology, Faculty of Medicine and Health, Örebro University, 701 85 Örebro, Sweden; 2 Clinical Epidemiology and Biostatistics, School of Medical Sciences, Campus Örebro University Hospital, Örebro University, 701 85 Örebro, Sweden; 3 Unit of Biostatistics, Institute of Environmental Medicine, Karolinska Institutet, 171 77 Stockholm, Sweden; 4 Department of Obstetrics and Gynecology, Skaraborgs Hospital Skövde, 541 85 Skövde, Sweden; 5 University Health Care Research Center, Faculty of Medicine and Health, Örebro University, 701 85 Örebro, Sweden

**Keywords:** Cesarean section, Surgical wound infection, Antibiotic prophylaxis, Costs and cost analysis

## Abstract

**Background:**

The proportion of pregnant women delivered by cesarean section has increased steadily during the past three decades. The risk of infection is 10-fold augmented after elective cesarean section compared to vaginal delivery. Antibiotic prophylaxis may reduce endometritis by 62% and superficial wound infection by 38% after elective cesarean section. International guidelines recommend antibiotic prophylaxis in elective cesarean section, but this procedure is not routinely followed in Sweden. Studies of costs of antibiotic prophylaxis in cesarean section show conflicting results and are based on substantially different incidence of postoperative infections. No study of costs of antibiotic prophylaxis in elective cesarean section in a Swedish or Nordic context has been pursued. The aim of this study was to investigate if antibiotic prophylaxis is cost-reducing in elective cesarean section in Örebro County, Sweden.

**Methods:**

All women undergoing elective cesarean in the Region Örebro County health care system during 2011–2012 were eligible for inclusion. Postoperative infections and risk factors for infections were registered. A hypothetical situation in which all participants had received antibiotic prophylaxis was compared to the actual situation, in which none of them had received antibiotic prophylaxis. The reduction in the risk of postoperative infections resulting from antibiotic prophylaxis was based on a meta-analysis. Costs for in-patient care of postoperative infections were extracted from the accounting system, and costs for out-patient care were calculated according to standard costs. Costs for antibiotic prophylaxis were calculated and compared with the cost reduction that would be implied by the introduction of such prophylaxis.

**Results:**

The incidences of deep and superficial surgical site infection were 3.5% and 1.3% respectively. Introduction of antibiotic prophylaxis would reduce health care costs by 31 Euro per cesarean section performed (95% credible interval 4–58 Euro). The probability of cost-saving was 99%.

**Conclusions:**

Antibiotic prophylaxis in elective cesarean section is cost-reducing in this health care setting. Our results indicate that the introduction of antibiotic prophylaxis in elective cesarean section can also be cost-saving in low infection rate settings.

*Trial registration* Ethical approval was given by the Regional Ethical Review Board in Uppsala (registration number 2013/484).

## Background

The proportion of pregnant women delivered by cesarean section has increased steadily during the past three decades [1]. The risk of infection is 10-fold augmented after elective cesarean section compared to vaginal delivery [2]. A large meta-analysis found that the incidence of endometritis after elective cesarean section was 7%, but the incidence varied remarkably (0–24%) in the included studies; the risk of superficial wound infection was 8.5%, with a similar variation among the studies [3]. In a Swedish study of primiparas undergoing elective cesarean section due to breech presentation or psychosocial indication, the incidence of endometritis was 3.2% and no superficial wound infection was found [4].

Obesity and diabetes increase the risk of postoperative infection by 2 and 1.4 times respectively, but the coexistence of both risk factors increases the risk 9-fold compared to women with neither [5]. Smoking increased the risk of postoperative infection by 2.7 times in an extremely obese population of women [6].

According to a meta-analysis, antibiotic prophylaxis reduces endometritis by 62% and superficial wound infection by 38% after elective cesarean section [7]. International guidelines recommend antibiotic prophylaxis in elective cesarean section [8], but this procedure is not routinely followed in Sweden [9].

Studies of costs of antibiotic prophylaxis in cesarean section show conflicting results. While some studies show antibiotic prophylaxis to be cost reducing [10–12] others demonstrate the opposite [13–15]. The majority of the studies consider either emergency cesarean section [13] or a mix of emergency and elective cesarean section [10, 12, 14]. Only two studies specifically consider costs of antibiotic prophylaxis in elective cesarean section, one from the United States [11] and one from China [15]. The American study showed a cost reduction of 2% of the total cost per cesarean section, corresponding to US$30 per cesarean section. In contrast, the Chinese study showed a 12%, or approximately US$147, increase in the cost of each cesarean section with the introduction of antibiotic prophylaxis. No study of costs of antibiotic prophylaxis in elective cesarean section in a Swedish or Nordic context has been conducted.

## Methods

### Aim

The aim of this study was to investigate the incidence of superficial surgical site infection and deep surgical site infection, and to analyze whether antibiotic prophylaxis is cost-reducing in elective cesarean section in Örebro County, Sweden. This county has around 270,000 inhabitants and is situated in central Sweden.

### Participation characteristics

All women undergoing elective cesarean section from 1 January 2011 to 31 December 2012 at the Departments of Obstetrics and Gynecology at Örebro University Hospital and Karlskoga Hospital were eligible for the study. The extent of the time period for inclusion was decided according to the yearly number of women delivered by elective cesarean section at the hospitals and the incidence of postoperative infections earlier described in a Swedish population [7]. Exclusion criteria were having received antibiotic prophylaxis or having been treated with prophylactic antibiotics (for example due to urinary tract anomaly), having left Örebro County after delivery or not having been followed up by the Region Örebro County health care system for any other reason, having been reoperated due to causes other than wound infection, and having undergone other major procedures during the cesarean section.

**Fig. 1:**
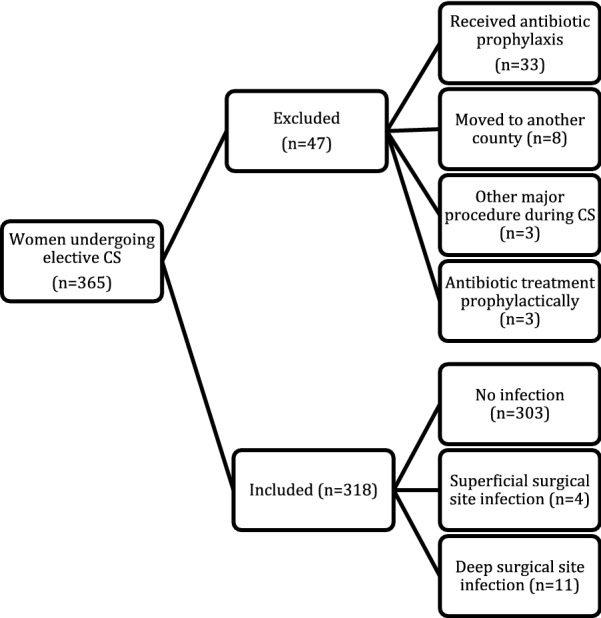
Excluded women, reasons for exclusion, included women, and incidence of surgical site infections. *CS* cesarean section

Of a total of 6871 women delivered in Örebro County between 2011 and 2012, 365 (5.3%) underwent elective cesarean section. Of these, 47 were excluded (Fig. 1). The main reason for exclusion was having received antibiotic prophylaxis, and another important reason was having moved out of the county. The mean age was 32.5 years, 24% were obese, 4% had diabetes mellitus, and 4% were smokers.

### Process

The women were identified through the search function in the Obstetrix medical record system (Siemens, version 2.14.02.200). Medical records from primary health care, maternity health care, specialist maternity health care, and obstetric in-patient care in Örebro County were studied. The following possible risk factors for infection were registered: smoking, any type of diabetes mellitus, obesity (BMI > 30 kg/m^2^), excessive perioperative hemorrhage (> 1000 ml), and postoperative thromboprophylaxis; the first three of these were extracted from maternity health care records and the others were extracted from obstetric in-patient care unit records. Postoperative infections were classified as superficial surgical site infection and deep surgical site infection developing up to 30 days postoperatively, according to the Centers for Disease Control definitions of nosocomial surgical infections [16]. Deep surgical site infection was considered equal to endometritis, which is the term used in the two meta-analyses cited previously [3, 7]. Urinary tract infections were excluded since there is no evidence they are preventable by antibiotic prophylaxis [7].

To minimize the risk of underreporting the incidence of postoperative infections, all health care visits during the first 30 days after each cesarean section were identified and studied. One woman with a deep surgical site infection and several women with superficial surgical site infections were treated in units other than the Departments of Obstetrics and Gynecology at the two hospitals.

### Intervention

All participants were assumed to receive antibiotic prophylaxis.

### Comparison

The same participants not receiving any antibiotic prophylaxis.

### Economic analysis

A health care perspective was used in the analysis, meaning that only health care costs were included. The intervention cost and the cost of infections developed during the first month after delivery were considered. The care costs preventable by an introduction of antibiotic prophylaxis were calculated assuming the same effect of antibiotic prophylaxis as in the meta-analysis cited earlier [7], namely, a relative reduction in the risk of endometriosis (0.62, or 95% CI 0.47–0.82) and of superficial wound infection (0.38, or 95% CI 0.24–0.61) after elective cesarean section. Finally, the care costs preventable by antibiotic prophylaxis were compared to the costs for administering antibiotics to all women undergoing elective cesarean section during the given time period.

Costs for antibiotic prophylaxis were obtained from the Swedish Medicines Compendium for health care professionals (FASS). Where prices were not fixed, costs were obtained from the pharmacy at Örebro University Hospital. The costs of administering antibiotic prophylaxis (ampicillin 2 g intravenously) included both material and personnel costs (the latter including salaries and payroll tax). Estimated time required was acquired from the head of the ward caring for women undergoing elective cesarean section at Örebro University Hospital, and the costs of material and personnel time were acquired from Örebro University Hospital accounts. It was assumed that two-thirds of an employee’s time is spent on patient care, and the rest on activities such as preparation, further education, meetings, and breaks.

Costs for the in-patient care of the postoperative infections were extracted from the Region Örebro County accounting system (ECOMED). Costs for all in-patient care are registered in this system according to a Swedish system called cost per patient (KPP), which is mainly used for debiting patient costs from other counties. The KPP encompasses all hospital health care in Sweden, and is used to produce national average costs for a certain treatment of a certain disease. Costs for out-patient care were calculated using the standard prices for out-patient visits that are used to debit patient costs from other counties.

Costs were calculated at the 2014 price level and expressed in Euro, transformed from Swedish Crowns using exchange rate 1 Euro = 9 Swedish Crowns. Overhead costs for the health authority were included in the costs for care of postoperative infections but not in the costs for administering antibiotic prophylaxis. To ensure comparability, proportions of overhead costs were estimated and then excluded from the costs of care for postoperative infections.

Three alternative assumptions were tested in a sensitivity analysis:

- Half of an employee’s time instead of 2/3 is spent on patient care.
- 15% of overhead costs instead of 9%.
- The effect of antibiotic prophylaxis is reduced according to the lower end of 95% confidence interval presented in the meta-analysis [7]; 47% instead of 62% for endometritis and 24% instead of 38% for superficial wound infection.

### Statistics analysis

Descriptive data are presented as absolute numbers and percentages. The risk of infection was calculated in a univariate analysis as odds ratios (ORs) with 95% confidence intervals (CIs). Version 22 of the IBM SPSS software package was used for the statistical calculation. Statistical uncertainty of the incidence of infections was calculated using release 11 of the STATA software package (STATA, TX, USA). Average cost reductions and corresponding 95% credible intervals as well as probability of cost-saving were estimated using a resampling method with replacement and 10,000 replications in version 3.2.2 of the R statistical software package [17].

## Results

The incidence of superficial surgical site infection was 1.3% and the incidence of deep surgical site infection was 3.5%, giving a 4.8% total incidence of surgical site infections (95% CI 2.7–7.7). Table 1 presents distribution of potential risk factors for infections and their correspondig Odds ratios. The only risk factor that proved to be statistically significant was smoking (OR 4.08; 95% CI 1.05–15.94).

**Table 1:** Risk factors for infections and odds ratios (ORs) with 95% confidence intervals (CIs) for surgical site infection

	Number	%	OR	95% CI	No information available
Smoking	13	4.0	4.08	1.05–15.94	3
Diabetes	12	3.8	1.90	0.27–13.16	0
Obesity (BMI > 30)	77	24	0.99	0.33–2.98	4
Excessive perioperative hemorrhage (> 1000 ml)	46	15	1.51	0.44–5.14	1
Thromboprophylaxis	143	45	1.42	0.53–3.82	0

*DSI* deep surgical site infection, *SSI* superficial surgical site infection

**Fig. 2:**
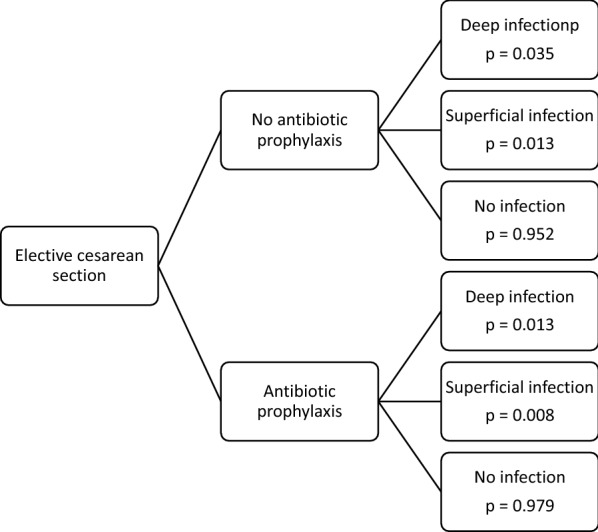
Risk of infection with and without antibiotic prophylaxis

Figure 2 presents the risk of infection with and without antibiotic prophylaxis.

The health care cost of surgical site infections was 27,307 Euro, consisting of 4560 Euro for superficial surgical site infections and 22,747 Euro for deep surgical site infections. Women subject to superficial surgical site infection were exclusively treated as out-patients, whereas most women subject to deep surgical site infection were mainly treated as in-patients. The costs for in-patient care were considerably higher than those for out-patient care (Table 2).

**Table 2:** Costs for postoperative infections

Patient number	Type of surgical site infection	Doctor visits in primary health care		Doctor visits in specialist care		In-patient care		Nurse visits in primary health care		Nurse visits in specialist care		Costs for antibiotic in out-patient care	Total cost
		Number	Cost	Number	Cost	Days	Cost	Number	Cost	Number	Cost		
1	Superficial	7	1045	2	509	0	0	17	952	0	0	111	2617
2	Superficial	0	0	2	355	0	0	0	0	1	54	28	437
3	Superficial	0	0	1	182	0	0	0	0	0	0	25	207
4	Superficial	3	784	1	228	0	0	4	224	0	0	63	1299
Sum SSI			1829		1274		0		1176		54	227	4560
5	Deep	0	0	0	0	4	2405	0	0	0	0	13	2418
6	Deep	1	261	0	0	0	0	0	0	0	0	14	275
7	Deep	0	0	4	887	3	3280	0	0	1	54	41	4262
8	Deep	0	0	4	887	11	5531	0	0	1	54	36	6508
9	Deep	0	0	1	177	0	0	0	0	0	0	48	225
10	Deep	0	0	4	729	0	0	0	0	0	0	50	779
11	Deep	0	0	1	182	0	0	0	0	0	0	37	219
12	Deep	0	0	0	0	2 + 3	2474	0	0	0	0	48	2522
13	Deep	0	0	0	0	2	594	0	0	4	144	24	762
14	Deep	0	0	0	0	2 + 3	3137	0	0	0	0	48	3185
15	Deep	0	0	0	0	3	1574	0	0	0	0	18	1592
Sum DSI			261		2862		18995		0		252	377	22747
Sum SI			2090		4136		18995		1176		306	604	27307

All costs are in Euros

*DSI* deep surgical site infection, *SI* surgical site infection, *SSI* superficial surgical site infection

Table 3 presents the cost of care preventable by antibiotic prophylaxis based on a primary estimation of 9% for overhead costs of in-patient care, while Table 4 presents the cost of administering antibiotic prophylaxis using a primary estimation that two-thirds of an employee’s time is spent on patient care. Based on these estimations, introduction of routine antibiotic prophylaxis would deliver a saving of 31.2 Euro (45.3 minus 14.1) per patient (95% credible interval 4–58 Euro). Probability of cost-saving was 99%.

**Table 3:** Health care costs of 318 women for the preventable proportion of surgical site infections based on a primary estimation of 9% overhead costs

Total cost for care of SSI	4560
Cost for care of SSI excluding overhead costs	4150
Proportion of costs of SSI preventable by antibiotic prophylaxis (38%)	1577
Total cost for care of DSI	22,747
Cost for care of DSI excluding overhead costs	20,698
Proportion of costs of DSI preventable by antibiotic prophylaxis (62%)	12,833
Total cost preventable by antibiotic prophylaxis	14,410
Total cost per women	45.3

All costs are in Euros

*DSI* deep surgical site infection, *SSI* superficial surgical site infection

**Table 4:** Costs to administer antibiotic prophylaxis

	Cost per woman
Personnel	7.1
Antibiotics	6.4
Other material	0.6
Total cost per woman	14.1

All costs are in Euros

In a sensitivity analysis, alternative estimations were made under the assumptions that the overhead costs of in-patient care are 15% of the total, half of an employee’s time is spent on patient care, and with lower effect of antibiotic prophylaxis. The cost of administering antibiotic prophylaxis using these estimations is presented in Table 4. Calculations based on these alternative estimations deliver savings of 15–29 Euro per patient, with a probability of cost-saving of 87–98% (Table 5).

**Table 5:** Sensitivity analysis based on estimations of overhead costs of 9% and 15% respectively and assumptions that two-thirds or half of an employee’s time is spent on patient care, respectively

Preventable infections	38% and 62%				24% and 47%			
Overhead excluded	9%		15%		9%		15%	
Time spent on patient care	2/3	1/2	2/3	1/2	2/3	1/2	2/3	1/2
Care costs that are preventable	45.3	45.3	42.3	42.3	33.7	33.7	31.5	31.5
Costs to administer antibiotic prophylaxis	14.1	16.4	14.1	16.4	14.1	16.4	14.1	16.4
Cost reduction per woman	31.2	28.9	28.2	25.9	19.6	17.3	17.4	15.1
Probability of cost saving	0.986	0.980	0.984	0.976	0.920	0.885	0.904	0.865

All costs are in Euros

*OH* overhead costs

## Discussion

This cost-minimization analysis shows that an introduction of antibiotic prophylaxis in elective cesarean section would reduce health care costs by 31.2 Euro per cesarean section (95% credible interval 4–58 Euro), and a probability of cost-saving of 99%. The incidence of postoperative surgical site infection was 4.8% in this material, comprising 3.5% deep surgical site infections and 1.3% superficial surgical site infections. The only risk factor showing a significantly elevated risk for postoperative infection was smoking (OR: 4.08; 95% CI 1.05–15.94).

This study shows that antibiotic prophylaxis in elective cesarean section is cost-reducing even in this context where the incidence of postoperative infections is low. Since the introduction of prophylactic antibiotics in elective cesarean section would imply less suffering for the women, and there is no evidence that the risk of antibiotic resistance is greater with antibiotic prophylaxis, this speaks in favor of an introduction in this health care context.

The economic favorability of antibiotic prophylaxis can be explained by the fact that it is a relatively simple measure in a woman already admitted for a surgical procedure. This can be compared to the high costs of postoperative infections, which can be explained by the expensive in-patient care that many of the women with deep surgical site infections are subject to.

The incidence of postoperative surgical site infections in this study is low in an international perspective. The incidences of deep surgical site infection and superficial surgical site infection were 3.5% and 1.3% respectively, which can be compared to 7% and 8.5% respectively in the Cochrane Library meta-analysis [3]. The incidence of deep surgical site infection is comparable to that in a Swedish study of 247 primiparas undergoing elective cesarean section due to psychosocial indication or breech presentation, which reported an incidence of endometritis of 3.2%; however, no superficial surgical site infection was found in that study [4].

Smoking was a significant risk factor for postoperative infection in this study. A PubMed search specifically on this subject revealed only one study examining smoking as a risk factor for infection in cesarean section [6].

The cost reduction of 31.2 Euro per cesarean section is comparable to the reduction of costs seen by Chelmow et al. [11], which was US$30 per cesarean section. Hong et al. on the other hand, found a cost-increase of ¥924, or approximately US$147, per cesarean section [15]. The incidence of postoperative infection is crucial for the cost-effectiveness of antibiotic prophylaxis in a certain setting since a high incidence of postoperative infections favors the cost-reducing effect of antibiotic prophylaxis, whereas a low incidence disfavors the effect. Chelmow et al. used an incidence of endometritis of 4.8% in their model, whereas the incidence found by Hong et al. was 1.2%, which can partly explain the difference seen in effect on costs. The incidence of endometritis in the present study is in between the two cited studies. Transfer of results of cost-effectiveness from one study to another context must thus be made with caution.

The study design we have chosen has some limitations. This is an observational study where we have used the reduced risk reported in a meta-analysis [7] to calculate the cost reduction.

The meta-analysis is based on a review of studies including a total of 15,000 women and extending over more than 40 years. The evidence was judged as moderate using the GRADE approach. The studies differed in many aspects, such as setting, antibiotic regimen, and risk of infection, but the authors found no evidence of statistically important heterogeneity in the effect of antibiotic prophylaxis. Studies including emergency and elective cesarean section were both included, but the effects of antibiotic prophylaxis in different subgroups of cesarean section were analyzed and reported separately. Based on this, we assume that the effect of antibiotic prophylaxis would be comparable in our setting.

The limited sample size of 318 women means that conclusions about the incidence of postoperative infections are uncertain. The total incidence of postoperative infection was 4.8% (95% CI 2.7–7.7). The confidence interval calculated implies that the true incidence might differ somewhat from what we found. This is of importance since our cost calculations are based on the incidence of postoperative infections.

The impact a postoperative infection can have on quality of life was also not taken into account in this study. As most postoperative infections are mild and of short duration, one might assume that the impact on quality of life in terms of quality-adjusted life years is small in the majority of patients.

There has been debate whether antibiotic prophylaxis administered preoperatively affects the fetus negatively. It is known that the first generation of cephalosporin (cefazolin) is transferred to the fetus [18], and hence some authors have argued that antibiotic prophylaxis should be administered after the umbilical cord has been clamped, in order to avoid unnecessary fetal exposure to antibiotics [19]. However, a meta-analysis by Hessen et al. showed no difference in neonatal outcome when administering antibiotic prophylaxis preoperatively compared to administering antibiotics after clamping the umbilical cord, whereas the risk of endometritis was significantly decreased [20].

There is no evidence regarding the extent to which antibiotic prophylaxis contributes to development of antibiotic resistance [7]. It is thus hard to make a scientific comparison between antibiotic prophylaxis and antibiotic treatment regarding the risk of antibiotic resistance. However, the issue of antibiotic prophylaxis is of great importance. When comparing the risks of development of antibiotic resistance, relevant factors include the efficiency of antibiotic prophylaxis, how many doses an antibiotic treatment includes, and the incidence of postoperative infections.

## Conclusions

This is the first study to show that antibiotic prophylaxis in elective cesarean section is cost-reducing in a Swedish and Nordic context. Specifically, antibiotic prophylaxis in elective cesarean section is cost-reducing with the incidence of postoperative infections and in the health care context of Örebro County, Sweden. Our results indicate that the introduction of antibiotic prophylaxis in elective cesarean section can also be cost-saving in low infection rate settings.

## References

[1] Vogel JP, Betrán AP, Vindevoghel N, Souza JP, Torloni MR, Zhang J (2015). Use of the Robson classification to assess caesarean section trends in 21 countries: a secondary analysis of two WHO multicountry surveys. Lancet Global Health..

[2] Burrows LJ, Meyn LA, Weber AM (2004). Maternal morbidity with vaginal versus caesarean delivery. Obstet Gynecol.

[3] Smaill F, Hofmeyr GJ (2002). Antibiotic prophylaxis for cesarean section. Cochrane Database Syst Rev..

[4] Larsson C, Saltvedt S, Wiklund I, Andolf E (2011). Planned vaginal delivery versus planned caesarean section: short-term medical outcome analyzed according to intended mode of delivery. J Obstet Gynaecol Can..

[5] Schneid-Kofman N, Sheiner E, Levy A, Holcberg G (2005). Risk factors for wound infection following cesarean deliveries. Int J Gynaecol Obstet.

[6] Alanis MC, Villers MS, Law TL, Steadman EM, Robinson CJ (2010). Complications of cesarean delivery in the massively obese parturient. Am J Obstet Gynecol.

[7] Smaill FM, Grivell RM (2014). Antibiotic prophylaxis versus no prophylaxis for preventing infection after cesarean section. Cochrane Database Syst Rev..

[8] Caesarean Section, NICE Clinical Guidelines, No. 132 (2011). National collaborating centre for women’s and children’s health (UK).

[9] SBU-rapport nr 200, Antibiotikaprofylax vid kirurgiska ingrepp, en systematisk litteraturöversikt, Statens beredning för medicinsk och social utvärdering; 2010. [SBU-report no 200, Antibiotic prophylaxis in surgical procedures, a systematic review, Swedish Agency for Health Technology Assessment and Assessment of Social Services; 2010].

[10] Bibi M, Megdiche H, Ghanem H, Sfaxi I, Nouira M, Essaidi H (1994). Antibiotic prophylaxis in a priori cesarean sections without a high risk of infection. Experiences of a Tunisian maternity department. J Gynecol Obstet Biol Reprod..

[11] Chelmow D, Hennesy M, Evantash EG (2004). Prophylactic antibiotics for non-laboring patients with intact membranes undergoing cesarean delivery: an economic analysis. Am J Obstet Gynecol.

[12] Rudge MV, Atallah AN, Peraçoli JC, Tristão Ada R, Mendonça Neto M (2006). Randomized controlled trial on prevention of postcesarean infection using penicillin and cephalothin in Brazil. Acta Obstet Gynecol Scand.

[13] Kristensen GB, Beiter EC, Mather O (1990). Single-dose cefuroxime prophylaxis in non-elective cesarean section. Acta Obstet Gynecol Scand.

[14] Mallaret MR, Blatier JF, Racinet C, Fauconnier J, Favier M, Micoud M (1990). Economic benefit of using antibiotic prophylactically in cesarean sections with little risk of infection. J Gynecol Obstet Biol Reprod.

[15] Hong F, Zhang L, Zhang Y, Sun W, Hong H, Xu Y (2016). Antibiotic prophylaxis to prevent postoperative infectious morbidity in low-risk elective cesarean deliveries: a prospective randomized clinical trial. J Matern Fetal Neonatal Med..

[16] Horan TC, Gaynes RP, Martone WJ, Jarvis WR, Emori TG (1992). CDC definitions of nosocomial surgical site infections, 1992: a modification of CDC definitions of surgical wound infections. Infect Control Hosp Epidemiol.

[17] Efron B (1981). Nonparametric estimates of standard error: the jackknife, the bootstrap and other methods. Biometrika.

[18] Fiore Mitchell T, Pearlman MD, Chapman RL, Bhatt-Mehta V, Faix RG (2001). Maternal and transplacental pharmacokinetics of cefazolin. Obstet Gynecol.

[19] Lamont RF, Sobel JD, Kusanovic JP, Vaisbuch E, Mazaki-Tovi S, Kim SK (2011). Current debate on the use of antibiotic prophylaxis for caesarean section. BJOG.

[20] Hessen M, Klöhr S, Rossaint R, Allegeaert K, Deprest J, Van de Velde M (2013). Concerning the timing of antibiotic administration in women undergoing caesarean section: a systematic review and meta-analysis. BMJ Open.

